# Efficacy and safety analysis of hypofractionated and conventional fractionated radiotherapy in postoperative breast cancer patients

**DOI:** 10.1186/s12885-024-11918-2

**Published:** 2024-02-06

**Authors:** Yongkai Lu, Beina Hui, Di Yang, Yi Li, Binglin Li, Luping Zhou, Lei Xu, Fengwen Tang, Wei Wang, Ruijuan Chen, Dongli Zhao

**Affiliations:** 1 Department of Radiation Oncology, The First Affiliated Hospital of Xi’an Jiaotong University, No.277, Yanta West Road, Xi’an, 710061 Shaanxi China; 2 Department of Radiation Oncology, Shaanxi Provincial Tumor Hospital, Affiliated Hospital of Xi’an Jiaotong University Health Science Center, Xi’an, China; 3 Department of Obstetrics and Gynecology, Xi’an Central Hospital, the Affiliated Hospital of Xi’an Jiaotong University, Xi’an, 710003 Shaanxi China; 4 Department of Radiation Oncology, Xijing Hospital, Air Force Medical University, Xi’an, 710003 China

**Keywords:** Breast cancer, Hypofractionated radiotherapy, Conventional fractionated radiotherapy, Meta-analysis

## Abstract

**Objectives:**

In this meta-analysis, we conducted a comparative analysis of the safety and efficacy of hypofractionated and conventional fractionated radiotherapy in individuals who had undergone surgery for breast cancer.

**Methods:**

This study involved a systematic and independent review of relevant research articles published in reputable databases such as PubMed, Embase, Cochrane Library, and Web of Science. Two investigators conducted the review, which included studies published up to January 3, 2023. The quality of the eligible studies was evaluated and data were extracted using Review Manager software 5.4 (RevMan 5.4) to calculate odds ratios (ORs) and 95% confidence intervals (CIs).

**Results:**

The analysis comprised 35 studies and encompassed a collective sample of 18,246 individuals diagnosed with breast cancer. We did not find a statistically significant disparity in efficacy between conventional fractionated (CF) radiotherapy and hypofractionated (HF) radiotherapy regarding local recurrence (LR; OR = 0.91, 95% CI: 0.76–1.09, *P* = 0.30), disease-free survival (DFS; OR = 1.20, 95% CI: 1.01–1.42, *P* = 0.03), and overall survival (OS; OR = 1.08, 95% CI: 0.93–1.26, *P* = 0.28). Concerning safety, there was no significant difference between the HF and CF regimens in terms of breast pain, breast atrophy, lymphedema, pneumonia, pulmonary fibrosis, telangiectasia, and cardiotoxicity. However, the HF regimen resulted in lower skin toxicity (OR = 0.43, 95% CI: 0.33—0.55, *P* < 0.01) and improved patient fatigue outcomes (OR = 0.73, 95% CI: 0.60 – 0.88, *P* < 0.01).

**Conclusions:**

Although there is no substantial difference in LR, DFS, OS, or many other side effects between the HF and CF regimens, the HF regimen reduces skin toxicity and relieves patient fatigue. If these two issues need to be addressed in clinical situations, the HF regimen may be a superior alternative to conventional radiotherapy in postoperative breast cancer patients.

**Supplementary Information:**

The online version contains supplementary material available at 10.1186/s12885-024-11918-2.

## Introduction

Breast cancer is the most common cancer that occurs in women worldwide. In 2020, female breast cancer surpassed lung cancer as the most commonly diagnosed cancer, with an estimated 2.3 million new cases [1]. Adjuvant radiation for breast cancer patients is associated with improved cancer-specific survival and a decreased chance of locoregional recurrence [2]. For many years, conventional fractionation (CF), which recommended 50 Gy/50.4 Gy over 25–28 sessions of 1.8–2 Gy per day, was the most popular standard dose of radiation therapy. This plan was formulated on the presumption that daily doses above 2 Gy may exacerbate the negative effects of the treatment [3]. However, the standard 5–6 weeks of radiotherapy is inconvenient for many patients, underlining the need for more cost-effective and comfortable treatments, particularly during the COVID-19 outbreak.

In recent years, hypofractionated radiation therapy (HFRT) has emerged as a viable substitute for conventional radiation therapy in the treatment of breast cancer patients [4]. Whelan et al. [5] demonstrated that ten years post-treatment, accelerated hypofractionation whole-breast irradiation was comparable to standard radiation therapy in terms of efficacy for women with invasive breast cancer who had undergone breast-conserving surgery with clear surgical margins and negative axillary lymph nodes. Subsequently, long-term randomized trials, such as the START A and START B trials, provided evidence that hypofractionated radiotherapy yielded equivalent outcomes to conventionally fractionated radiotherapy [6–8]. Based on this, the guidelines from the European Society of Medical Oncology suggest a moderate hypofractionation regimen comprising 15–16 fractions of 3 Gy each [9]. Nevertheless, researchers are not limited to treatment regimens that are only moderately fractionated. The 5-year findings of the FAST-Forward trial, which were released in 2020 [10], are expected to result in a future increase in hypofractionated treatments consisting of only five fractions [11]. The employment of hypofractionated regimens for breast cancer radiation therapy has been supported by extensive randomized controlled trials.

As mentioned earlier, certain developed nations in Europe and North America have carried out extensive randomized controlled trials encompassing large sample sizes and extended durations. However, other countries and regions, including Belgium, China, Taiwan, Australia, and Korea, have only disclosed regional results, and other tests were only recently registered [12–16]. Thus, to provide broader guidance for clinical practice, it is necessary to conduct a comprehensive meta-analysis of the latest results from a variety of regions to determine the differences in efficacy and safety between the hypofractionated (HF) regimen and the conventional fractionated (CF) regimen in breast cancer radiotherapy. To address this need, we conducted a meta-analysis of contemporary controlled studies and retrospective studies to assess overall survival, recurrence rates, and various toxicity indicators after hypofractionated radiotherapy in breast cancer patients.

## Methods

### Search strategy

The authors searched the PubMed, Embase, Web of Science, Cochrane Library, and Clinicaltrials.gov databases to find relevant articles published before January 3, 2023. Only peer-reviewed publications related to human adults were included and there were no language restrictions. The following search strategy was used: (breast cancer) AND (hypofractionated fractionation OR hypofractionation) AND (conventional fractionation OR conventional). Additionally, the authors manually searched reference lists to locate any citations that the computer-assisted search may have overlooked. Any discrepancies were settled through discussion between the two authors. This research followed the recommendations of Preferred Reporting Items for Systematic Review and Meta-Analysis (PRISMA) [17].

### Study selection

One researcher (RJC) compiled a list of potentially pertinent papers by reviewing the citations that were revealed during the literature search. The entire text was examined if the applicability of a study could not be ascertained from only the title or the abstract. A second researcher (BLL) independently reviewed all texts for potential inclusion and disputes were settled through discussion.

The inclusion criteria included: (1) conventional fractionation regimens of less than 2 Gy per day in the control group and hypofractionation regimens of 2–5 Gy per day in the experimental group; (2) retrospective, prospective, and randomized controlled studies were evaluated for inclusion. The exclusion criteria included non-human data, lack of raw data, and incomplete reports. If duplicate publications used the same patient cohort, the study with the most complete data was included.

### Data extraction and quality assessment

The necessary data from eligible studies were extracted independently by two researchers (LPZ and LX) using standardized forms. Inconsistencies were addressed through discourse, with the involvement of a third team member (FWT), if necessary. The data extraction form contained the following information: first author, publication year, age, sample size, clinical tumor stage, outcome indicators, dose fractionation scheme, cohort characteristics and size, study design, and inclusion and exclusion criteria. To evaluate the risk of bias in the retrospective studies, we applied the Newcastle–Ottawa Scale (NOS) [18], which comprised three dimensions: selection, comparability, and outcome. On an overall scale from 0 to 9, four points were awarded for selection, two for comparability, and three for outcomes. Studies scoring at least 6 points were deemed high quality [19]. Additionally, the modified Jadad scale was employed to evaluate the quality of randomized controlled studies, with scores of 1–3 being low quality and scores of 4–7 reflecting high-quality studies [20].

### Statistical analysis

Statistical pooling was conducted using RevMan software version 5.4, which was developed by Cochrane Collaboration, Oxford, UK. The effect indicator chosen for the measurement data analysis was the odds ratio (OR), along with a 95% confidence interval (CI). The assessment of heterogeneity across trials was conducted using the Cochrane Q test and the* I*^2^ statistic, which provided the percentage of the total variability attributable to heterogeneity rather than random error [21]. In instances where the *P*-value of the Q test exceeded 0.10 and the *I*^2^ value was less than 50%, a fixed-effects model was employed to analyze data that exhibited non-significant heterogeneity [22, 23]. In cases of significant heterogeneity in the data, a random-effects model was employed. Additionally, a sensitivity analysis was conducted to assess the potential impact of a single study on the overall evaluation. This involved the iterative removal of one study at a time and pooling the remaining trials. Moreover, a funnel plot was created to assess potential publication bias in the literature. When the points within the funnel plot exhibit a symmetrical distribution on either side of the central dashed line and tend to cluster around the center, there is a low likelihood of publication bias. Otherwise, there is a higher likelihood of publication bias.

## Results

### Identified studies

**Fig. 1:**
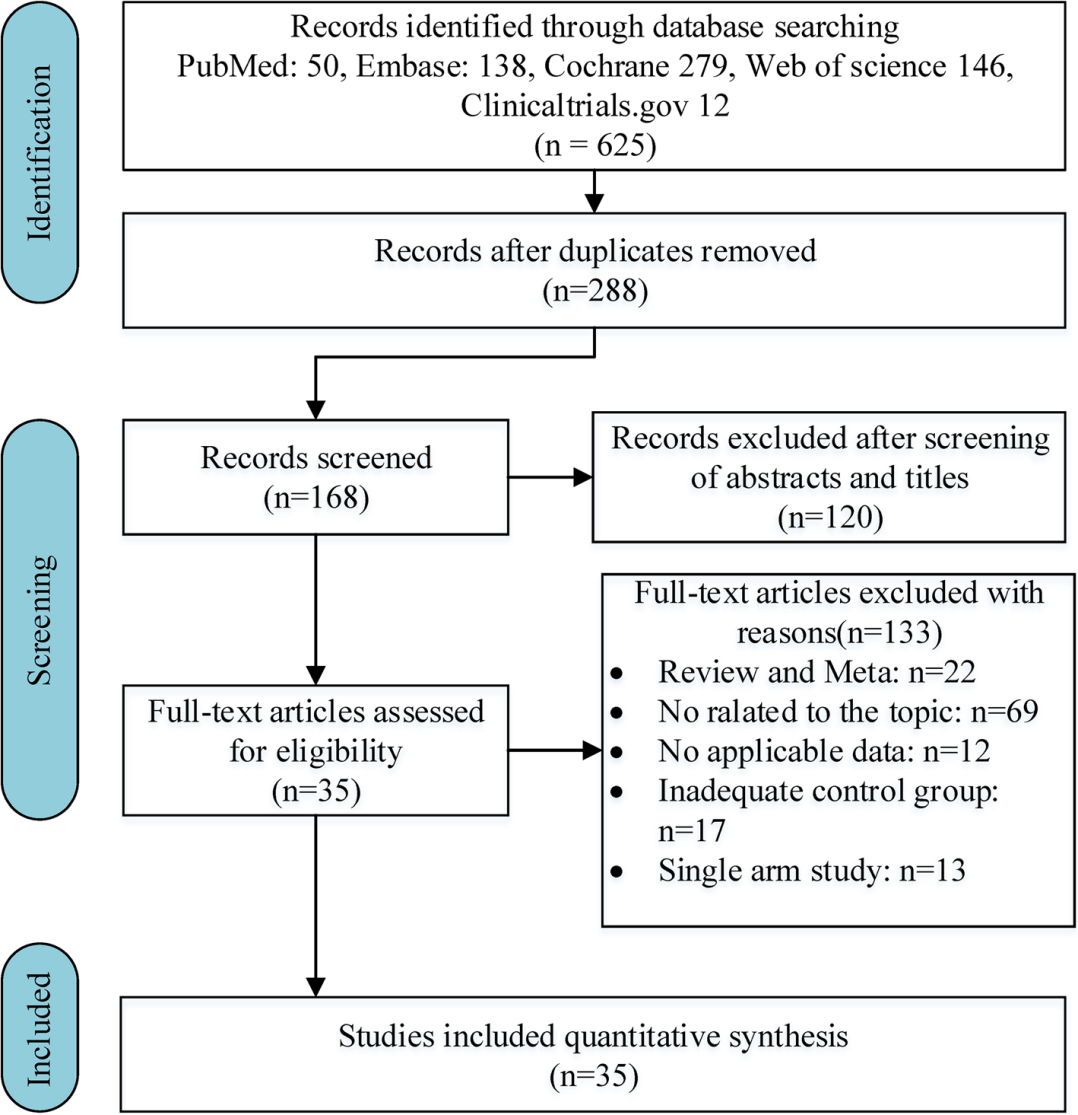
Flow chart of the search process for the meta-analysis

After eliminating 337 duplicate articles, an initial search of the multiple databases described above yielded 288 articles. Subsequently, by evaluating titles and abstracts, 165 ineligible papers were disregarded. Following a full-text review, 35 eligible articles were evaluated for design and quality. Figure 1 depicts the complete study selection procedure.

### Study characteristics

This paper involved a comprehensive analysis of 35 studies [5, 8, 14, 16, 24–54] comprising a total of 18,246 patients who had been diagnosed with breast cancer. The sample consisted of 13 randomized controlled trials with a Jadad score exceeding 4 and 22 retrospective studies with a Newcastle–Ottawa Scale score of 6 or higher. Table 1 provides a summary of the baseline information for the 35 included studies. It is noteworthy that the two studies conducted by Simona et al*.* [46, 47] shared identical sample sizes and baseline characteristics. However, they examined distinct outcome indicators and were thus not regarded as duplicate studies for this investigation.

**Table 1:** Characteristics of the included studies

**First author (Year of publication)**	**Total Patients (HF/ CF)**	**Clinical stage**	**Age range**	**Dose-fractionation scheme**		**Study type**	**NOS or Jadad score**	**Reported outcomes**
				**HF**	**CF**			
Andrew 2016 [24]	197 (56/141)	DCIS	—	42.2–42.6 Gy/16f	45–50 Gy/25f	Retrospective	7	Ipsilateral recurrence rate; cosmetic effect
Biragitte 2020 [25]	1854 (917/937)	pT1-2, pN0-1	> 40	40 Gy/15f	50 Gy/25f	RCT	6	Breast depression, breast atrophy, breast pain, cosmetic effect, hyperpigmentation, edema, etc
Boon 2022 [14]	1608 (777/831)	DCIS	> 18	42–45 Gy/16f	50 Gy/25f	RCT	6	Time to local recurrence, overall survival, various toxicities, cosmetic effects, quality of life, etc
Brady 2022 [26]	331 (246/85)	T1-T2	29–87	40.05 Gy/15f	50 Gy/25f	Retrospective	8	Skin toxicities, etc
Chadha 2012 [27]	124 (50/74)	Tis, T1, T2	29–88	40.05 Gy/15f	46.8 Gy/26f	Retrospective	7	Skin toxicity, breast pain, breast edema, fatigue, and hematologic side effects, etc
Christopher 2012 [28]	1335 (1083/252)	T1-T2, N0, M0	48–66	42.5–44 Gy/16f	45–50 Gy/25	Retrospective	7	Local relapse, distant relapse, etc
Chuang 2021 [29]	718 (359/359)	pT1-2, pN0, M0	26–90	40-42 Gy/15–16 f	46-50 Gy/23–25 f	Retrospective	8	Ipsilateral recurrence rate, overall survival, acute skin toxicity, etc
Fabian 2005 [30]	129 (65/64)	pT1-2, pN0-1	—	2.5 Gy 4 × /week to 55 Gy	2.0 Gy 5 × /week to 55 Gy	Retrospective	6	Breast pain, breast fibrosis, breast atrophy, telangiectasia, lymphedema, etc
Felice 2017 [31]	120 (58/62)	invasive breast cancer	39–82	42.5 Gy/16f	50 Gy/25f	Retrospective	6	Acute skin toxicity, cardiac and lung toxicity
Grazia 2013 [32]	339 (198/141)	pT1-2, pN0-1	22–86	44 Gy/16f	50 Gy/25f	Retrospective	6	Acute skin toxicities
Hany 2012 [33]	107 (66/41)	invasive breast cancer	25–68	40 Gy/15f 45 Gy/17f	50 Gy/25f	Retrospective	7	Erythema, fibrosis, Pain, telangectesia, arm oedema, pigmentation, etc
Hou 2015 [34]	80 (40/40)	pT1-2N0-1M0	≥ 18	43.2 Gy/18f	45 Gy/25f	Retrospective	8	Locoregional recurrence, acute and advanced skin reactions, aesthetic outcome, etc
Joanne 2013 [8]	2215 (1110/1105)	pT1-3a, pN0-1, M0	—	41.6 Gy/13f 39 Gy/13f	50 Gy/25f	RCT	7	local–regional relapse, distant relapse, disease-free survival, overall survival, normal tissue effects, etc
Julie 2020 [35]	161 (79/82)	node-negative invasive carcinoma	—	42.56 Gy/16f	50 Gy/25f	RCT	5	Acute skin reactions, quality of life, etc
King 2020 [36]	1148 (532/615)	DCIS	≥ 18	42.5 Gy/16f	50 Gy/25f	RCT	7	Local recurrence, overall survival, cosmetic outcome, radiation toxicity, etc
Kitwadee 2021 [37]	73 (37/36)	T1-3N0-1M0	33–76	43.2 Gy/16f	50 Gy/25f	Retrospective	6	Disease free survival, overall survival and toxicity
Kumar 2018 [45]	101 (47/54)	—	—	40 Gy/15f	50 Gy/25f	Retrospective	8	Acute and chronic toxicities, locoregional response, etc
Lee 2016 [39]	758 (379/379)	pT1-2, pN0-1a	26–81	39 Gy/13f	50.4 Gy/28f	Retrospective	6	Ipsilateral breast tumor relapse, distant metastasis, overall survival, etc
Leonard 2020 [40]	140 (70/70)	DCIS/T1/T2	19–84	40.05 Gy/15f	50 Gy/25f	RCT	6	Acute radiation-induced skin toxicity, etc
Maiti 2020 [41]	222 (120/102)	T0-T4, N0-N3	25–70	40 Gy/15f	50 Gy/25f	Retrospective	7	Locoregional tumour recurrence and normal tissue toxicities
Mishra 2016 [42]	100 (56/44)	IB- IIIC	—	42.4 Gy/16f	50 Gy/25f	Retrospective	6	Local failure, distant failure, skin Toxicity, dysphagia, pulmonary, lymphoedema, etc
Rastogi 2017 [43]	100 (50/50)	—	21–66	42.72 Gy/16f	50 Gy/25f	RCT	4	Toxicity, tolerability, and locoregional control
Reshma 2015 [44]	2309 (578/1731)	Tis-T4	—	single > 2 Gy	single ≤ 2 Gy	Retrospective	9	Dermatitis, pain, fatigue, and other common toxic effects
Sanjal 2018 [38]	60 (30/30)	T3, T4 Nx, N0 to N3	> 45	40.05 Gy/15f	50 Gy/25f	Retrospective	7	Pulmonary, cardiac, dermatological, toxicities and lymphoedema. feasibility option, local control
Simona 2015 [46]	287 (138/149)	Tis-T2, N0-N1a, M0	≥ 40	42.56 Gy/16 f	50 Gy/25f	RCT	6	Acute dermatitis, hyperpigmentation, fatigue, breast pain, pruritus etc
Simona 2018 [47]	287 (138/149)	TisT2N0-N1M0	≥ 40	42.56 Gy/16 f	50 Gy/25f	RCT	6	Functional status, local recurrences, distant metastasis, breast pain, cosmetic etc
Tomo 2008 [48]	443 (66/377)	Tis-T4	19–81	40 Gy/16 f	50 Gy/25f	Retrospective	7	Acute radiation dermatitis and pneumonitis
Vassilis 2016 [49]	117 (87/30)	T2-T4	33–78	42.56 Gy/16 f 48.3 Gy/21 f	50 Gy/25f	Retrospective	6	Acute and late skin toxicity
Volker 2016 [50]	266 (121/145)	Tis-T4	—	40.05 Gy/15 f	50 Gy/25f	Retrospective	6	Skin toxicity
Wang 2019 [51]	820 (406/414)	T3-T4	18–75	43.5 Gy/15 f	50 Gy/25f	RCT	7	Locoregional recurrence, overall survival, disease-free survival, and acute and late radiation toxicities
Wang 2020 [16]	734 (368/366)	T1-2N0-3	—	43.5 Gy/15 f	50 Gy/25f	RCT	7	Local Relapse, survival outcomes, toxicity and cosmesis
Weng 2021 [54]	287 (138/149)	DCIS or Tis-T2, N0-N1a, M0	≥ 40	42.56 Gy/16 f	50 Gy/25f	RCT	6	Breast pain, cosmesis, etc
Whelan 2010 [5]	455 (235/220)	invasive carcinoma	—	42.5 Gy/16 f	50 Gy/25f	RCT	7	Local Recurrence, and skin toxicity
Xu 2018 [52]	114 (83/31)	T0-3N0-1	—	40.05 Gy/15 f	50 Gy/25f	Retrospective	7	Skin toxicities
Zhao 2017 [53]	107 (53/54)	pT1-2, pN0-1, and pMx	—	42.56 Gy/16 f	50 Gy/25f	Retrospective	7	Local recurrence, distant metastasis, cosmetic and delayed toxic effects

*CF* conventional fractionation, *HF* Hypofractionation, *NOS* Newcastle–Ottawa Scale, *DCIS* Ductal carcinoma in situ, *RCT* Randomized controlled trials

### Efficacy

**Fig. 2:**
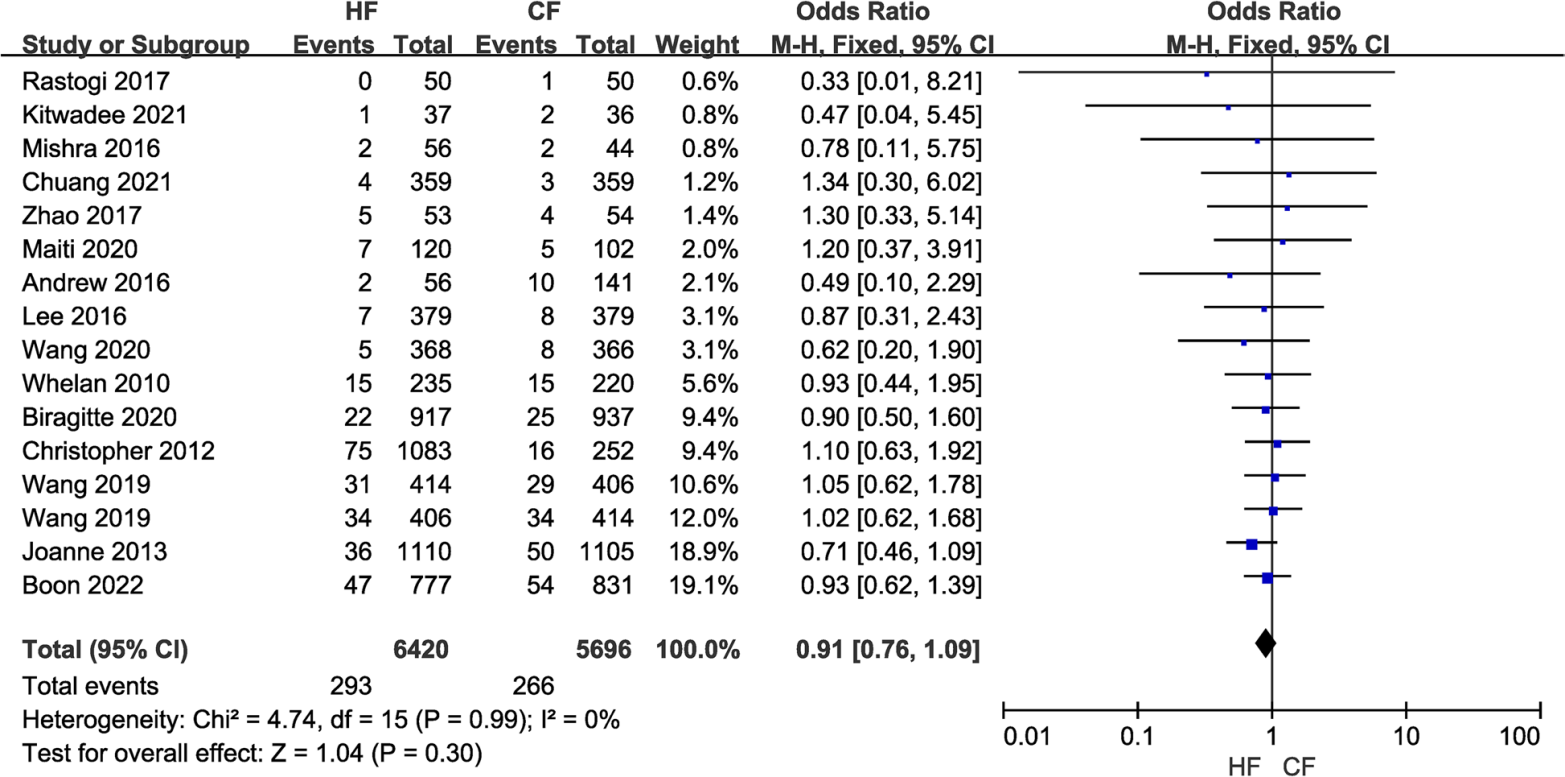
Forest plot of local recurrence rate between the HF group and CF group

**Fig. 3:**
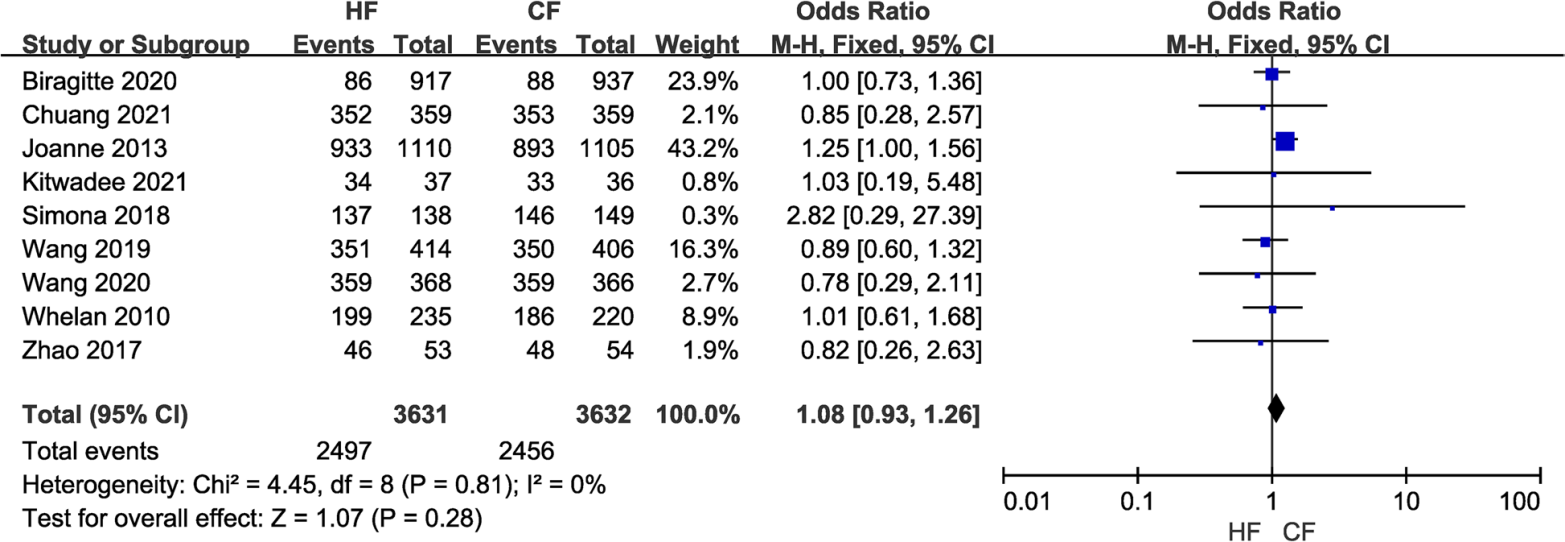
Forest plot of overall survival between the HF group and CF group

**Fig. 4:**
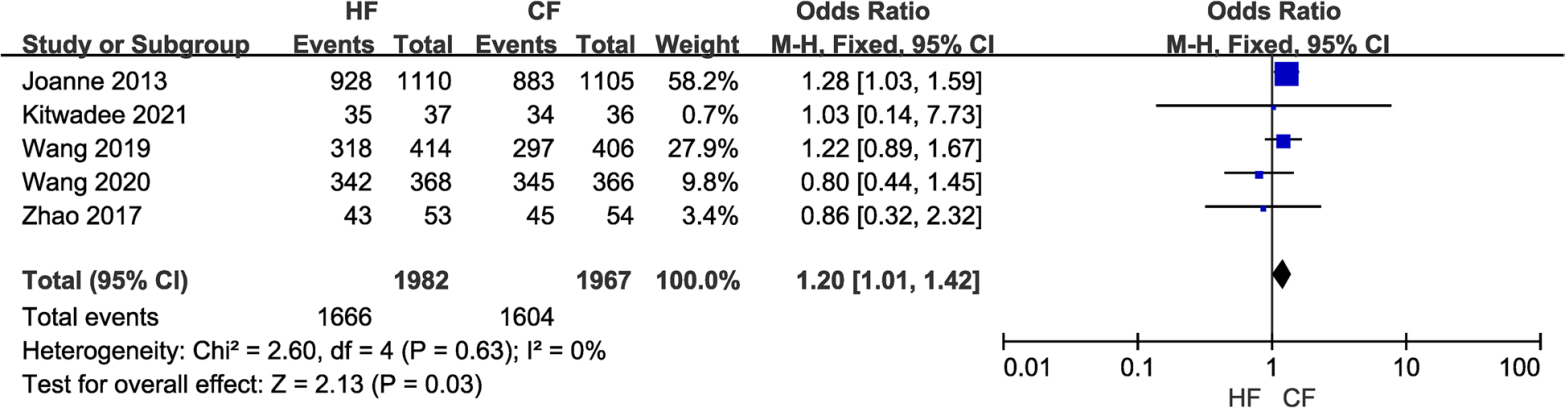
Forest plot of disease-free survival between the HF group and CF group

Efficacy comprises three indicators: local recurrence rate, overall survival rate, and disease-free survival rate. Data from a total of 12,116 breast cancer patients in 16 studies were included in the study of local recurrence rates. A fixed-effects model was chosen because of the low heterogeneity (*I*^2^ = 0) between studies. Pooled results showed no difference in local recurrence (LR) rates between the control and the experimental groups (OR = 0.91, 95% CI: 0.76–1.09, *P* = 0.30; Fig. 2). Moreover, the overall survival (OS) study contained the data of 7,263 breast cancer patients from nine investigations. Because of the minimal heterogeneity (*I*^2^ = 0) among trials, a fixed-effects model was adopted. Overall survival (OS) did not differ between the HF and CF groups, according to the pooled data (OR = 1.08, 95% CI: 0.93–1.26, P = 0.28; Fig. 3). Additionally, data on disease-free survival (DFS) were taken from five articles that assessed 3,949 people. Due to the insignificant between-study heterogeneity (*I*^2^ ≤ 50%, *P* > 0.10), the fixed-effects model was used. The combined data revealed no distinction between the HF group and CF group (OR = 1.20, 95% CI: 1.01–1.42, *P* = 0.03; Fig. 4). For each of the three data sets, sensitivity analyses were conducted, and no study with excessive heterogeneity altered the final aggregated results.

## Safety

The dissimilarities in safety between the two regimens were assessed using nine indicators related to toxicity and side effects, namely breast pain, breast atrophy, skin toxicity, lymphoedema, pneumonia, lung fibrosis, telangiectasia, fatigue, and cardiac events.

**Fig. 5:**
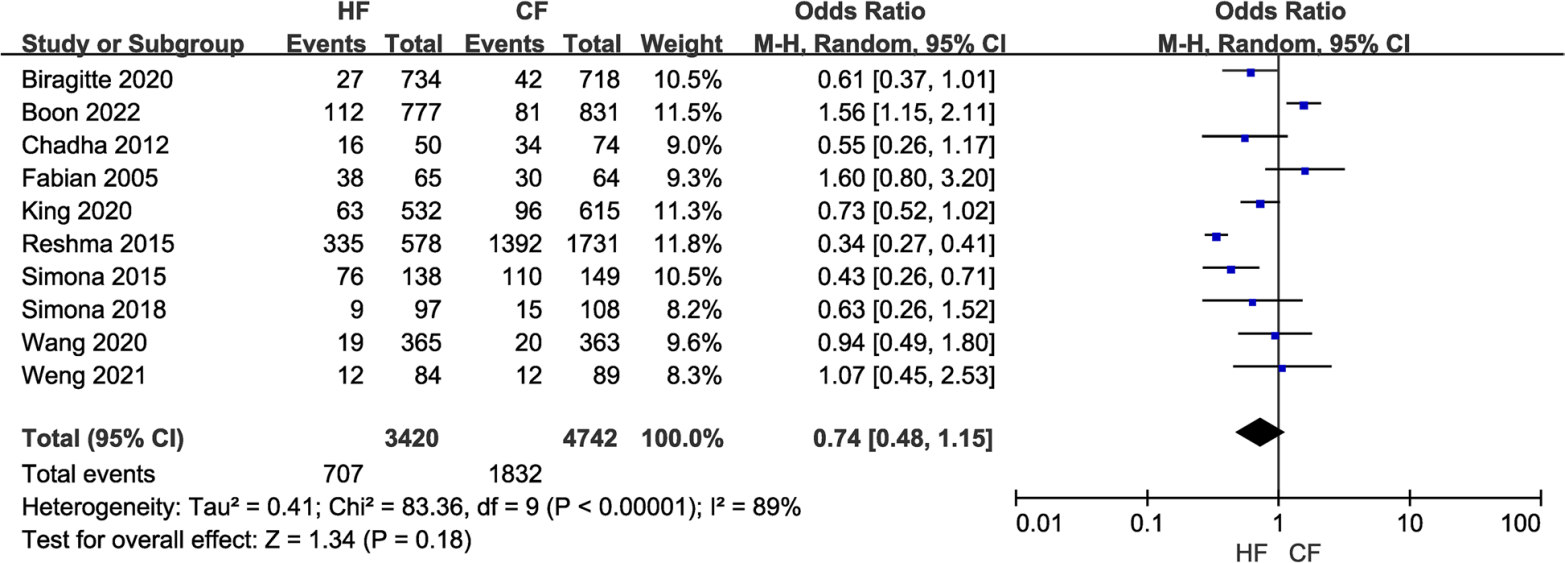
Forest plot of the incidence of breast pain between the HF and CF groups

**Fig. 6:**
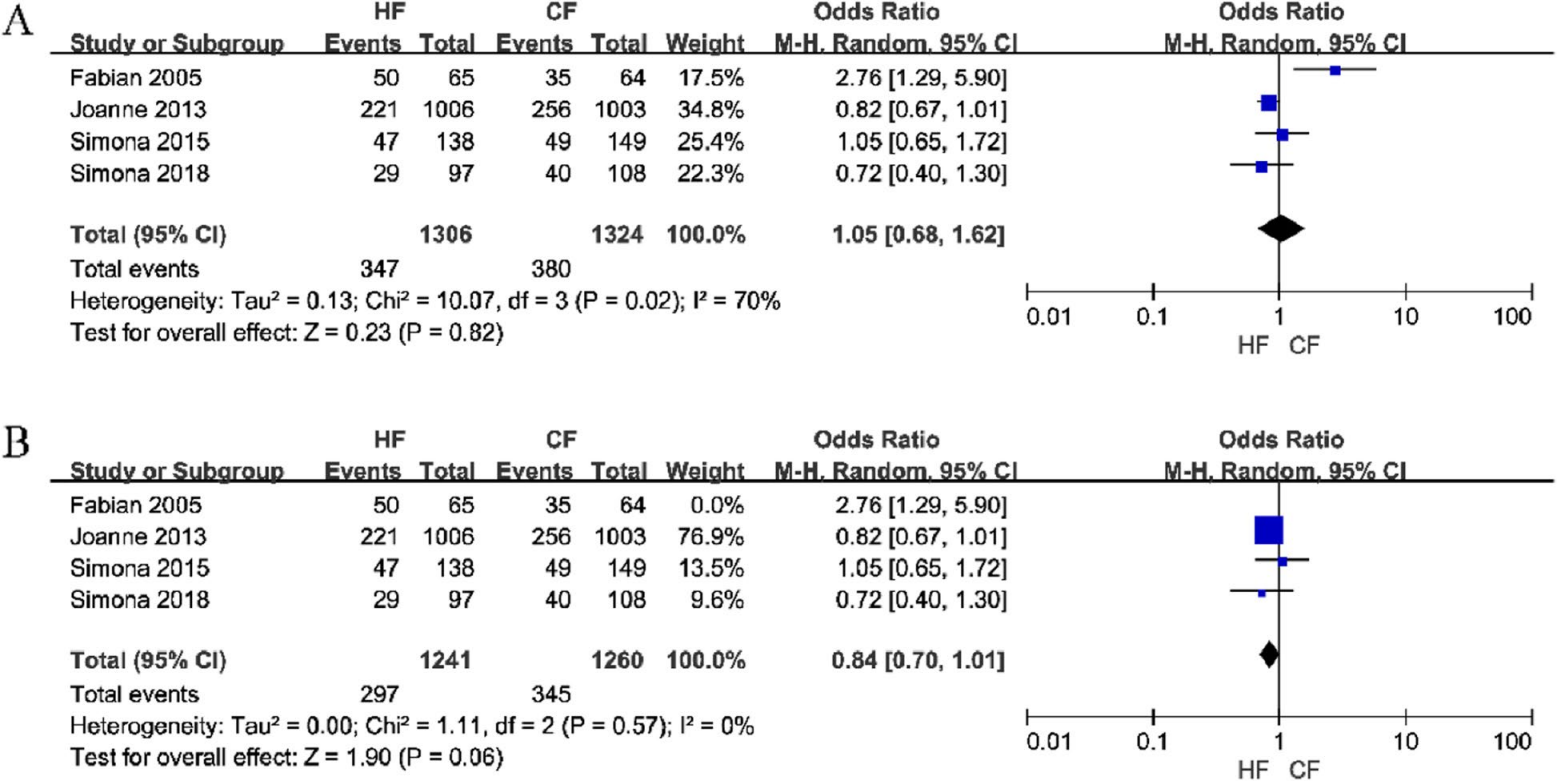
Forest plot of breast atrophy between the HF group and CF group (**A** is the summary result of all studies, **B** is the result after sensitivity analysis)

**Fig. 7:**
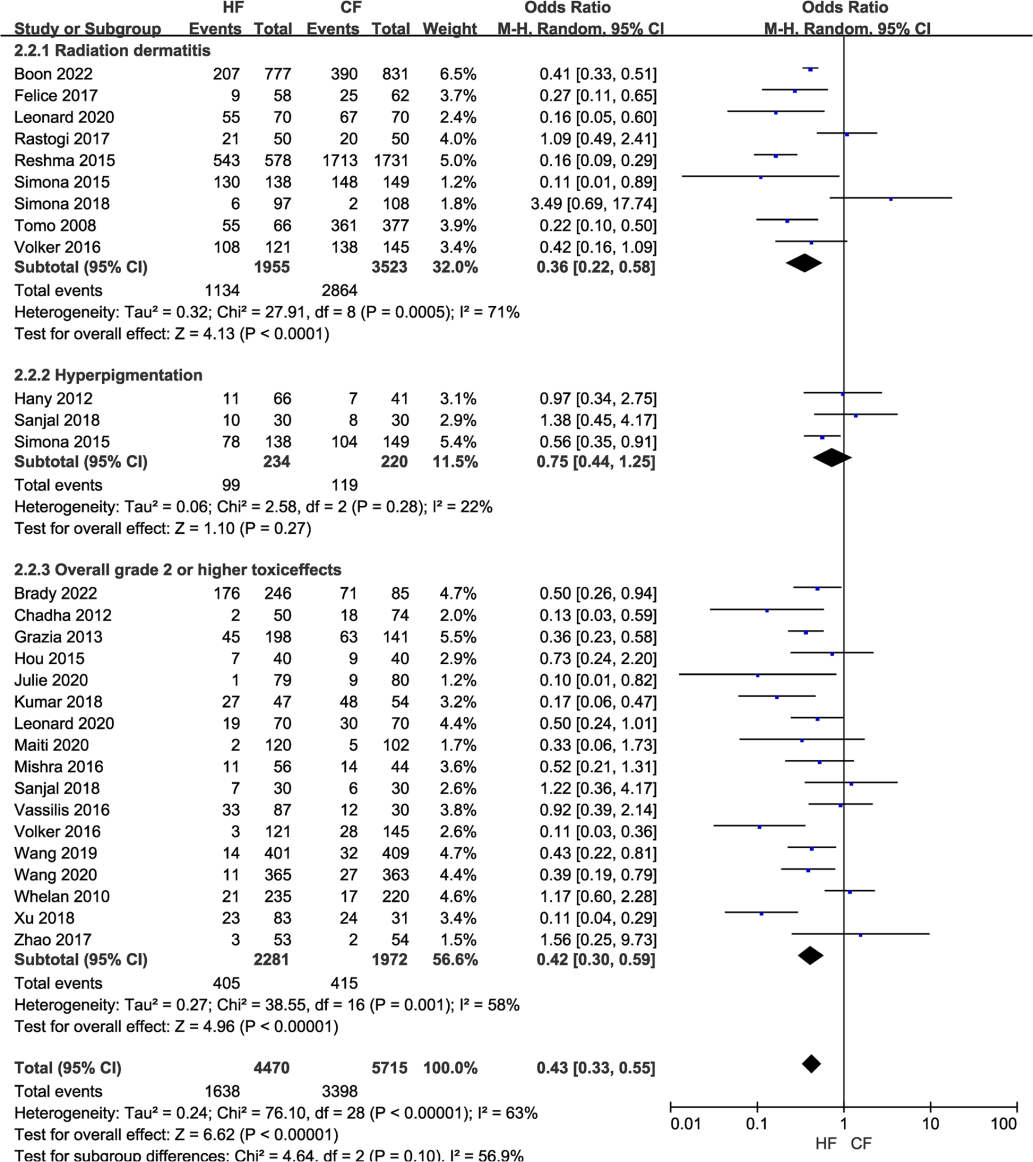
Forest plot of skin toxicities between the HF group and CF group

**Fig. 8:**
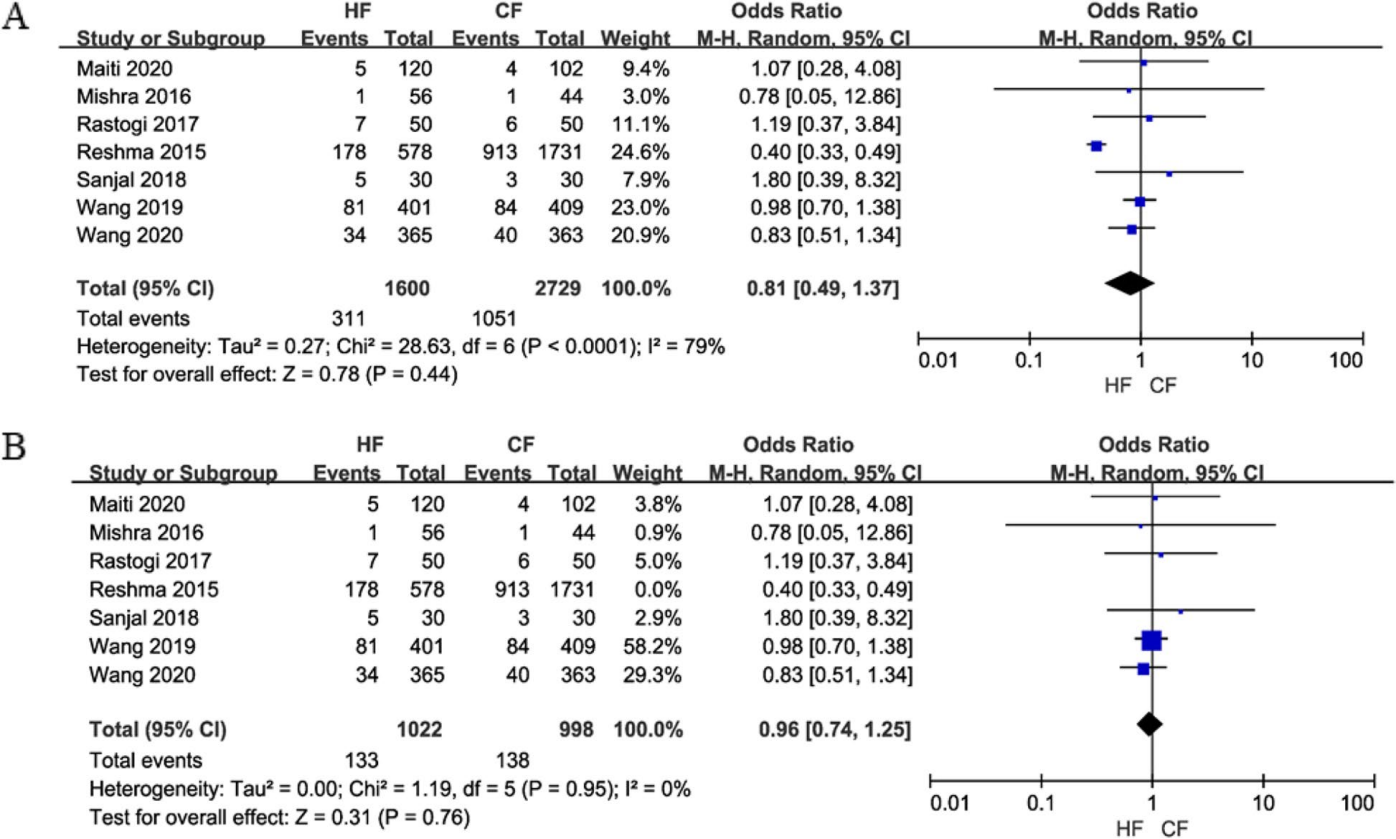
Forest plot of lymphedema between the HF group and CF group (**A** is the summary result of all studies, **B** is the result after sensitivity analysis)

**Fig. 9:**
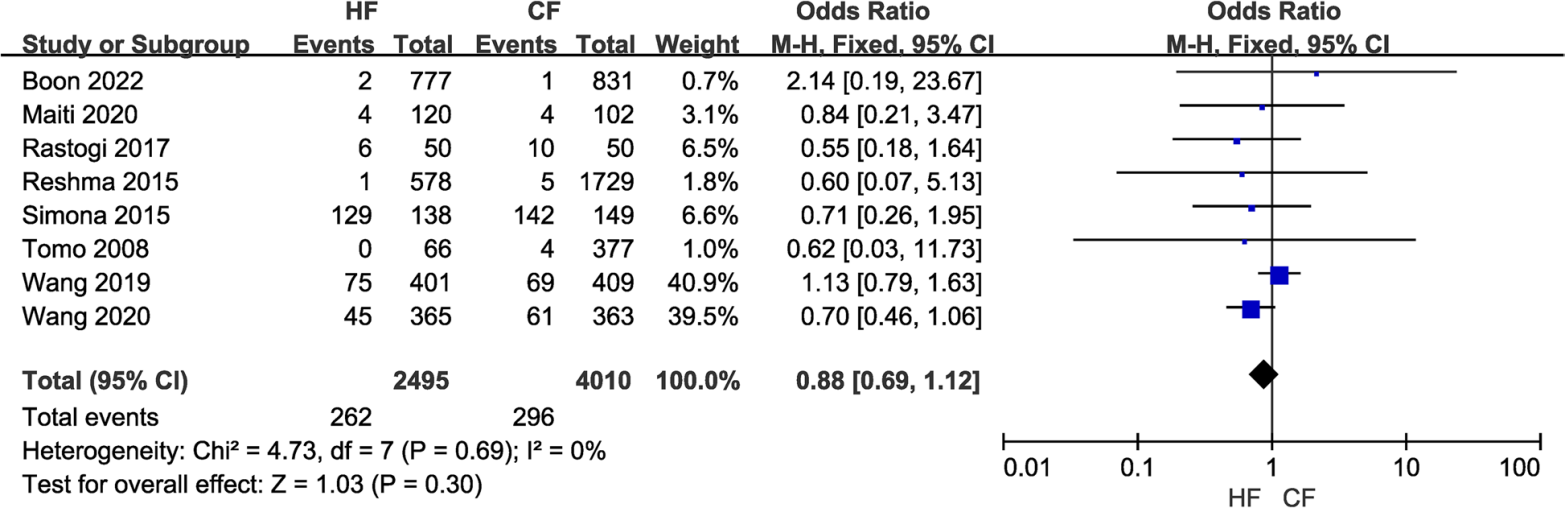
Forest plot of pneumonia between the HF group and CF group

**Fig. 10:**
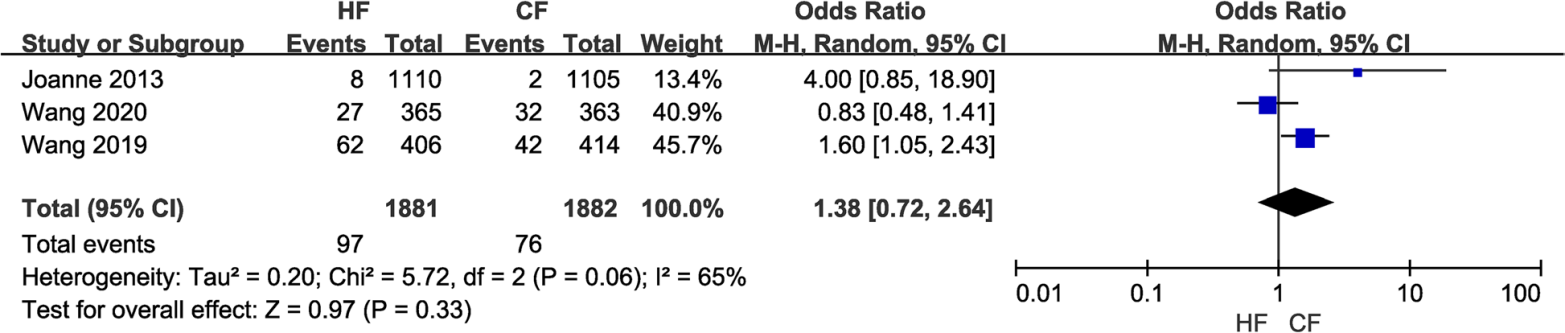
Forest plot of lung fibrosis between the HF group and CF group

**Fig. 11:**
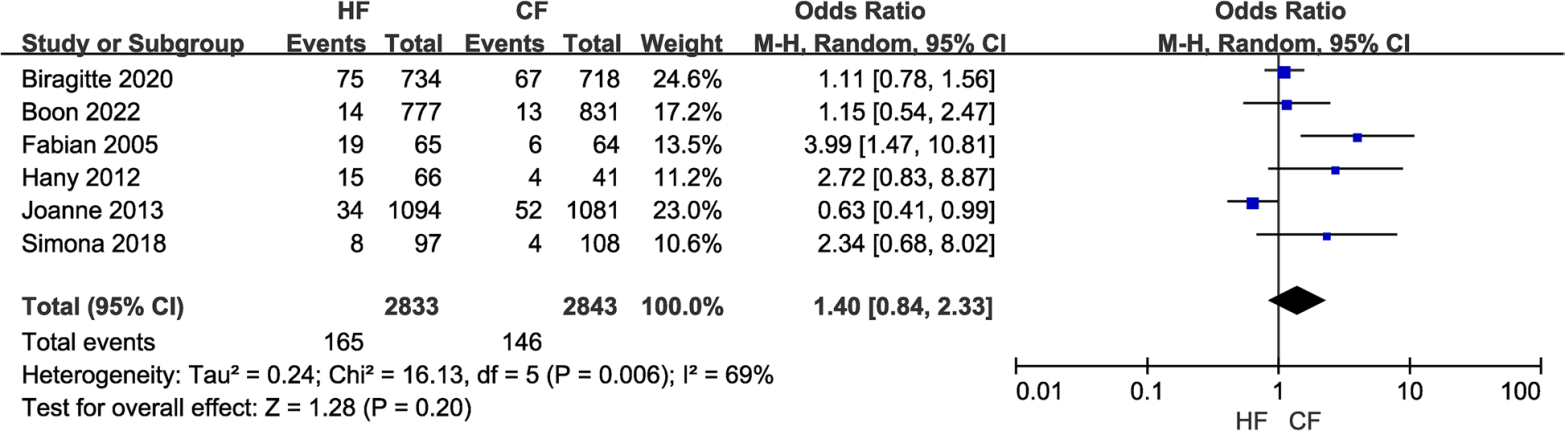
Forest plot of telangiectasia between the HF group and CF group

**Fig. 12:**
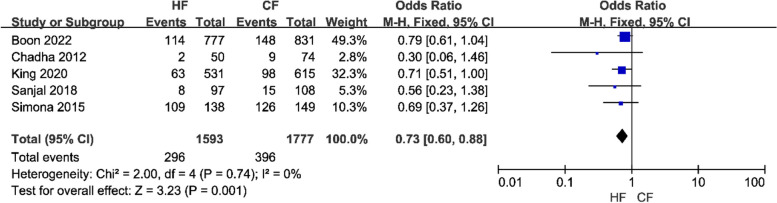
Forest plot of fatigue between the HF group and CF group

**Fig. 13:**
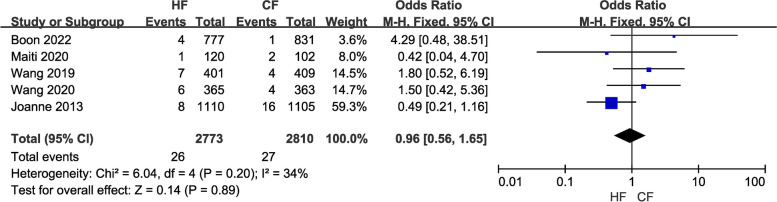
Forest plot of cardiac events between the HF group and CF group

1. Ten studies containing 8,162 participants reported on breast pain in patients after various treatment regimens. Because of the large heterogeneity (I2 = 89%) among studies, a random-effects model was applied. The results of the pooled analysis did not show any significant differences between the HF and CF groups (OR = 0.74, 95% CI: 0.48–1.15, *P* = 0.18; Fig. 5). The results did not change after performing a sensitivity analysis excluding one study at a time.

(2) Adverse events related to breast atrophy were addressed in four studies, which enrolled a total of 2,630 patients. A random-effects model was chosen for the analysis and the results showed no difference between the two fractionation regimens in causing breast atrophy in patients (OR = 1.05, 95% CI: 0.68–1.62, *P* = 0.82). A sensitivity analysis revealed that the heterogeneity decreased from 70 to 0% after excluding Fabian's [30] study, but the conclusion did not change (Fig. 6). The possible reasons for this occurrence are considered in the Discussion section.

(3) The investigation of cutaneous adverse reactions encompassed a cohort of 10,185 individuals across 25 research studies. Among the studies analyzed, radiation dermatitis was reported in 5,478 patients across nine studies, hyperpigmentation was reported in 454 patients in three studies, and skin toxicity of grade 2 or higher was reported in 4,253 patients from 17 studies. The combined pooled analysis showed that the HF regimen was superior in reducing skin toxicity (OR = 0.43, 95% CI: 0.33—0.55, *P* < 0.01), and the results did not change after sensitivity analysis. Furthermore, upon analyzing the three subgroups, the HF group exhibited superiority in two indicators, namely radiation dermatitis (OR = 0.36, 95% CI: 0.22—0.58, *P* < 0.01) and skin toxicity of level 2 or higher (OR = 0.42, 95% CI: 0.30—0.59, *P* < 0.01). However, there was no discernible difference between the HF and CF groups concerning the hyperpigmentation indicator (OR = 0.75, 95% CI: 0.44—1.25, *P* = 0.27; Fig. 7).

(4) Research on lymphedema was conducted on 4,329 participants in seven papers. Because of the large heterogeneity (I2 = 79%) among the studies, a random-effects model was chosen. The results of the meta-analysis showed that the two regimens posed similar risks of causing lymphedema in patients, with no significant differences (OR = 0.81, 95% CI: 0.49—1.37, *P* = 0.44). After a sensitivity analysis, the heterogeneity changed from 79 to 0% but this did not change the results, and the incidence of lymphedema was similar between the two regimens (OR = 0.96, 95% CI: 0.74—1.25, *P* = 0.76; Fig. 8).

(5) A comparative investigation was carried out on 6,505 patients across eight studies to examine the incidence of pneumonia following radiotherapy administered using the two distinct regimens. There was little heterogeneity (I2 = 0) among the studies, so a fixed-effects model was chosen for the meta-analysis. The results revealed no statistically significant difference between the two regimens regarding the development of pneumonia in patients (OR = 0.88, 95% CI: 0.69—1.12, *P* = 0.30; Fig. 9). The results of the sensitivity analysis did not affect the overall results.

(6) Three studies with a total of 3,763 patients reported on the occurrence of pulmonary fibrosis. A random-effects model was applied for pooled analysis and the results showed no significant difference in the incidence of pulmonary fibrosis between the HF and CF groups (OR = 1.38, 95% CI: 0.72—2.64, *P* = 0.33; Fig. 10).

(7) To examine the effects of the two radiotherapy regimens on telangiectasia occurrence, six studies with a total of 5,676 patients were included. A meta-analysis utilizing a random-effects model revealed no significant difference between the two treatment protocols in their propensity to induce capillary dilation among patients (OR = 1.40, 95% CI: 0.84—2.33, *P* = 0.20; Fig. 11). The results of the sensitivity analysis did not alter the outcome.

(8) Five studies examined fatigue in patients following radiotherapy. Because there was little variation among the studies, a fixed-effects model was used for the meta-analysis. The results revealed that the HF regimen lowered patient tiredness (OR = 0.73, 95% CI: 0.60 – 0.88, *P* < 0.01; Fig. 12). The sensitivity analysis results did not affect the outcome.

(9) Five studies involving 5,583 patients analyzed the incidence of cardiac events. Since no significant heterogeneity was identified (I2 ≤ 50%, *P* > 0.10), a fix-effects model was employed to calculate the pooled data. Regarding the incidence of adverse cardiac events, the data revealed no significant difference between the two regimens (OR = 0.96, 95% CI: 0.56 – 1.65, *P* = 0.89; Fig. 13). There was also no difference between the two regimens after the sensitivity analysis was conducted.

### Publication bias

**Fig. 14:**
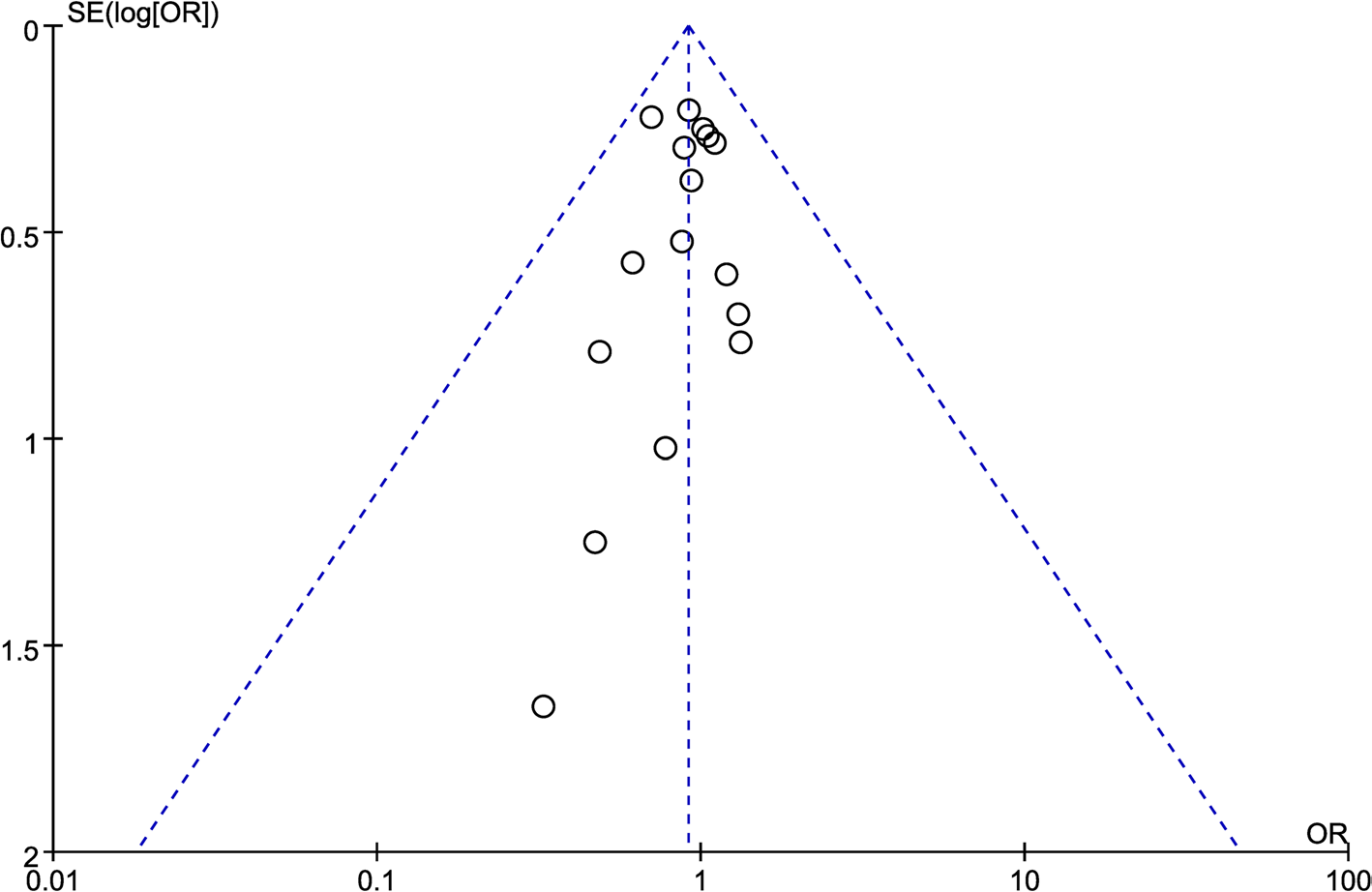
The funnel plot of the local recurrence rate

If at least ten papers were included in the meta-analysis, publication bias was assessed using a funnel plot, and tests for funnel plot asymmetry were performed. The funnel plot of the local recurrence rate (Fig. 14) indicates a symmetrical distribution of point estimates on both sides, with a concentration in the middle, thereby revealing no indication of publication bias. Funnel plots for other indicators are shown in the Supplementary material.

## Discussion

According to the 2022 Chinese Society of Clinical Oncology (CSCO) guidelines for the treatment of breast cancer, 50 Gy/25 sessions of conventional irradiation or 40–42.5 Gy/15–16 sessions of hypofractionated irradiation are recommended for patients whose target area includes only the affected whole breast [55]. The CSCO guidelines present a wider range of applicability than the ASCO guidelines [56]. Specifically, the HF regimen may be chosen if the treatment goal includes the entire afflicted breast. Furthermore, when considering the patient's healthcare and medical provisions, the HF option should also be selected. Nonetheless, there has been further research on the feasibility of HF therapy, with numerous clinical trials or trial protocols published in 2022 alone that compare the two treatment regimens. These studies examine the efficacy of the two approaches following breast reconstruction [15], non-low-risk ductal carcinoma in situ [14], or for patients necessitating regional lymph node irradiation [13]. Findings indicated that the validity of the data on HF was comparable to CF concerning local control, survival, and recurrence. However, HF exhibited a comparative advantage over CF concerning its association with a lower incidence of adverse events.

The meta-analysis in this study examined a total of 13 randomized controlled trials (RCTs) and 22 retrospective studies. The results indicated that there were no statistically significant differences between the HF and CF regimens regarding LR, OS, or DFS. Concerning safety, we observed no significant differences between the two regimens for adverse effects such as breast pain, breast atrophy, lymphedema, pneumonia, pulmonary fibrosis, capillary dilation, and cardiac events. However, compared to the CF treatment regimen, the HF regimen presented certain benefits such as decreased incidence of skin toxicity (including radiation dermatitis and grade 2 + skin toxicity) and reduced levels of patient fatigue.

The study on efficacy encompassed three metrics, namely LR, OS, and DFS. Before this study, three meta-analyses reported on pooled LR and OS [57–59]. The results of our research align with their findings, indicating that there were no notable disparities between the two treatment protocols regarding LR and OS outcomes. Furthermore, our research findings also reveal that the HF and CF schemes exhibit comparable outcomes with respect to DFS. The results we observed can be clarified through the lens of radiobiological principles. Besides, estimations of the biological effects of different radiation therapy schedules can be accomplished using a linear quadratic formula. This formula is based on several factors, including the quantity of radiation administered per day, the frequency of treatment, the dose of the treatment period, and a constant specific to the tissue endpoint known as the α/β ratio [60]. The α/β ratio exhibits a lower value for tissue that responds slowly, such as late fibrosis effects in normal tissue. Conversely, tissue that proliferates rapidly, including certain tumors, exhibits higher α/β ratios. The prevailing consensus is that the α/β ratio of neoplastic tissue typically falls within the range of 8–10. The CF protocol operates on the premise that breast cancer exhibits a lower sensitivity to alterations in fractionated doses compared to normal tissue. As a result, the administration of 2 Gy per fraction with a cumulative dosage of 50 Gy safeguards healthy tissue from potential harm [61]. However, investigations have revealed that the α/β value for breast cancer is substantially lower than the generally accepted tumor α/β value of around 4, with a range of 0.75–5.01 [62]. Furthermore, Haviland et al*.* [8] and Yarnold et al*.* [63] discovered that normal breast tissue had an α/β value of around 3.4, implying that the sensitivity of breast cancer tissue to dose partitioning was comparable to that of normal tissue. Based on these theories, HF protocols are appropriate when applied to breast cancer patients. The primary goal of HF is to efficiently eliminate tumors while minimizing hazardous side effects on normal tissue, as well as reducing the number of treatments and the cost burden on patients. As a result, the evidence in this study supports the viability of HF in the clinical management of breast cancer.

The incidence of toxic side effects reflects the safety of various radiotherapy regimens. In 2011, Lundstedt et al. [64] studied the risk factors for developing persistent breast pain after radiotherapy for breast cancer. The study included age at treatment, time since treatment, time since chemotherapy, photon energy, differentiation size, incremental volume, local radiotherapy, axillary surgery, overweight, and smoking factors. They ultimately concluded that only age and time since treatment were associated with the development of breast pain. The HF regimen with a fraction dose of 2.4 Gy was not related to the occurrence of breast pain, unlike the CF regimen with a 2.0 Gy fraction dose. The results of this study effectively support these findings. However, a more detailed explanation for breast pain may involve biological and psychological interaction. There is a belief that women experience cessation of ovarian function, leading to the onset of menopause, often occurring around the age of 50. Postmenopausal hormonal alterations have a significant impact on breast tissue, leading to a notable decrease in estradiol levels compared to the premenopausal stage. These variations may affect how the tissue reacts [64]. Regarding breast atrophy, we also found no statistically significant differences between the two protocols. However, a sensitivity analysis revealed that there was significant heterogeneity in the study of Fabian et al*.* [30]. The source of the heterogeneity is probably because the authors listed the same total dose of 55 Gy for both regimens, but the actual overall dose for the HF regimen reached 62 Gy, with individual fractions of 2.0 Gy. This ultimately led to the conclusion that the CF regimen was superior in reducing breast atrophy. When designing the HF regimen, the authors deviated from the current mainstream approach of fixing the total dose at 50 Gy and then converting to the HF regimen. Additionally, the small sample size was another possible cause of heterogeneity.

The toxicity that results from radiation therapy for breast cancer may lead to severe skin reactions. It may induce pain and potentially result in lasting skin damage, thereby necessitating temporary or permanent discontinuation of treatment. While variations in toxicity rates were observed among the trials that were assessed in this investigation, our findings reveal a reduced incidence of acute dermal toxicity after using HF. Despite the generally favorable outcomes, we cannot state that HF always reduces skin reactions in patients. The rationale behind this is the scarcity of studies with robust methodology regarding cutaneous toxicity, coupled with the multifactorial nature of the final skin response. This can be influenced by diverse variables including patient body mass index, breast volume, chemotherapy protocol, maximum dose to the breast, and varying boost administrations, among others [44, 65, 66]. The preeminent research substantiating the efficacy of HF is derived from the 2020 investigation conducted by Schmeel et al. [40]. The research team employed a combination of subjective physical assessments and objective skin spectroscopy measurements to evaluate skin reactions in both patient groups. The findings indicated that the HF regimen resulted in a decrease in the occurrence of dermatitis, erythema, and hyperpigmentation in patients. Nevertheless, it should be noted that the sample size in this study was limited. As such, further clinical trials are still required to definitively validate the advantages of HF.

Lymphedema is an observable medical condition that arises due to compromised lymphatic circulation. Adjuvant radiotherapy has been identified as a primary risk factor for its onset [67]. The findings of this study indicate that there was no discernible distinction between the two radiotherapy protocols in terms of lymphedema incidence among patients. The study conducted by Reshma et al. [44] exhibited strong heterogeneity, as demonstrated by the sensitivity analysis. This heterogeneity could be attributed to the lack of specificity in the administered HF and CF regimens, which were constrained by the uniform 2 Gy dosage. Furthermore, the dissimilarity in the number of samples utilized in the HF and CF treatments could be a factor in the manifestation of heterogeneity. Empirical data suggest that the irradiation of internal mammary lymph nodes and axillary lymph nodes during radiotherapy is associated with an elevated likelihood of lymphedema. Some clinicians proposed that the implementation of axillary reverse mapping, which involves the injection of technetium-99 into the breast and blue dye into the arm at risk, could potentially decrease the occurrence of lymphedema [68]. This aids the preoperative differentiation of axillary lymphatic drainage in the breast from that in the ipsilateral arm. However, the available data do not yet provide sufficient support for this claim [67].

Furthermore, the combined outcomes of the four adverse events examined in this investigation, namely pneumonia, pulmonary fibrosis, telangiectasia, and adverse cardiac events, revealed no significant statistical variance between the two treatment protocols. Due to the proximity of the breast to the lung, clinicians have expressed concern regarding radiation pneumonitis as a potential side effect. According to recent research, the development of pulmonary toxicity is influenced by several factors, including the type of radiation therapy energy utilized, the application of RT in the ipsilateral breast, the volume of 20 Gy received in the ipsilateral lung, the average dose administered to the ipsilateral lung. Pulmonary fibrosis is an irreversible disease and radiation-induced pulmonary fibrosis usually appears 6–12 months after radiotherapy [69]. Mechanistically, the initial stages of fibrogenesis following irradiation can be viewed as a wound-healing reaction. There is a rapid increase in the expression of pro-inflammatory cytokines, including tumor necrosis factor-α (TNFα), interleukins 1 and 6 (IL1 and IL6), and numerous growth factors within the affected tissue. Chemokines are secreted molecules that stimulate the recruitment of inflammatory cells from the neighboring tissue into the irradiated area. The precise mechanisms behind the interactions among the numerous proteins implicated in the fibrogenic process remain poorly understood [70]. Additionally, the administration of radiotherapy for breast cancer treatment may result in the exposure of the heart to radiation, potentially leading to adverse cardiac effects. According to Darby et al*.* [71], the exposure of the heart to ionizing radiation during radiotherapy for breast cancer is associated with an elevated risk of ischemic heart disease in the future. The escalation is commensurate with the mean heart dosage, commences within a few years of exposure, and endures for a minimum of twenty years. Contemporary research affirms that cardiac adverse effects correlate with the mean cardiac dose, the patient's respiratory exercise administration, and the radiotherapy modality [72]. However, there is insufficient evidence to substantiate the association with dose fractionation protocols. Cancer treatment-induced fatigue, commonly referred to as cancer-related fatigue (CRF), is a prevalent adverse effect, particularly among individuals undergoing breast cancer treatment. This study revealed that the selection of the HF regimen led to a reduction in fatigue following treatment, compared to alternative regimens. The study in question was conducted with limited sample size and revealed no notable impact of the graded separation regimen on fatigue and overall quality of life. Conversely, patients who underwent chemotherapy before radiotherapy exhibited a noticeable decrease in fatigue response [73].

The search we performed in this paper was thorough and the studies we considered featured high-quality RCTs and retrospective investigations, which enhanced the dependability of the results. Compared to prior meta-analyses on the same topic [57–59], our sample size and the research measures for side effects were larger, and we included ductal carcinoma in situ (DCIS) patients for the first time. Specifically, the sample size of Andrade et al. [59]was restricted to six studies, while the study conducted by Zhou et al*.* [58] comprised a relatively small number of research indicators. Therefore, additional verification is required to ascertain the quality of the evidence. Patients diagnosed with DCIS were excluded from the study by Gu et al*.* [57] due to data limitations, inadequate subgroup analyses, and the absence of sensitivity analyses.

Our study, however, has certain drawbacks. Due to a paucity of data, the subgroup analysis was inadequate. Concerning patient tumor staging, the meta-analysis was not particularly rigorous. Additionally, some salient factors such as the usage of boosters, systemic medication, and stratified follow-up time were not further stratified for analysis.

## Conclusions 

The findings of our investigation indicate that among breast cancer patients who have undergone surgery, both HF and CF treatment regimens produce consistent outcomes regarding LR, OS, and DFS. Furthermore, both treatment protocols can be deemed to be generally safe. Nevertheless, HF exhibits superior outcomes in relation to skin toxicity and fatigue. No significant variations were observed between the two treatment protocols concerning breast pain, breast atrophy, lymphedema, pneumonia, pulmonary fibrosis, telangiectasia, and cardiac toxicities. The safety and effectiveness of HF have been subject to a certain degree of scrutiny. Nevertheless, this treatment has yet to be fully implemented in clinical settings and requires further refinement.

### Supplementary Information

**Additional file 1.**

**Additional file 2.** Funnel plot for other indicators.

## Data Availability

All data generated or analysed during this study are included in this published article and its sup- plementary information files.
