# Supplementary material for: Efficacy and safety analysis of hypofractionated and conventional fractionated radiotherapy in postoperative breast cancer patients
Source: BMC Cancer. 2024 Feb 6;24:181. doi: 10.1186/s12885-024-11918-2 (PMC10845660; doi:10.1186/s12885-024-11918-2)

**Funnel plot for other indicators**

**
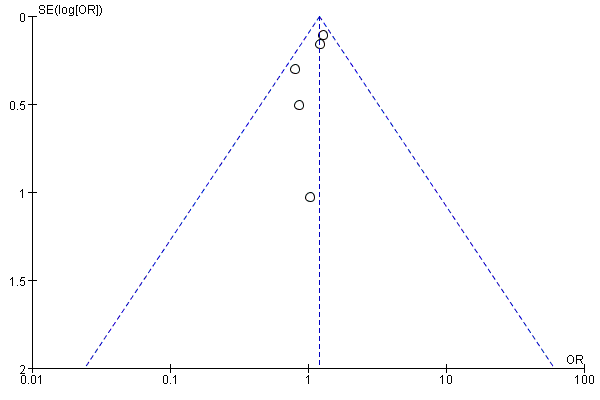

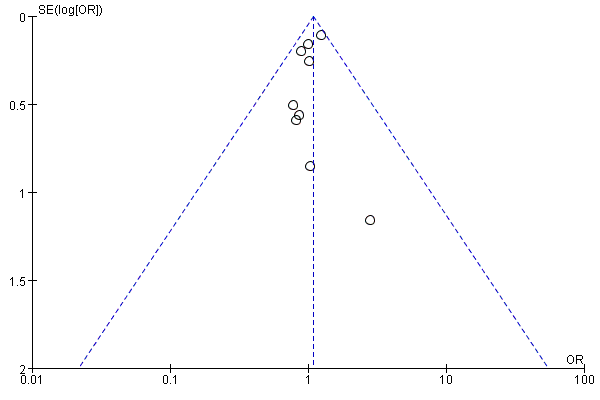
**

The funnel plot of the local recurrence rate
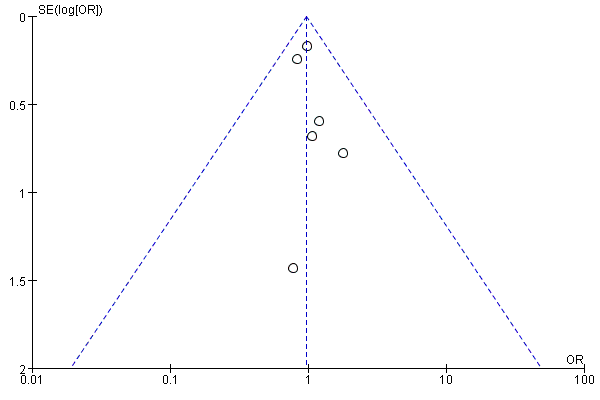

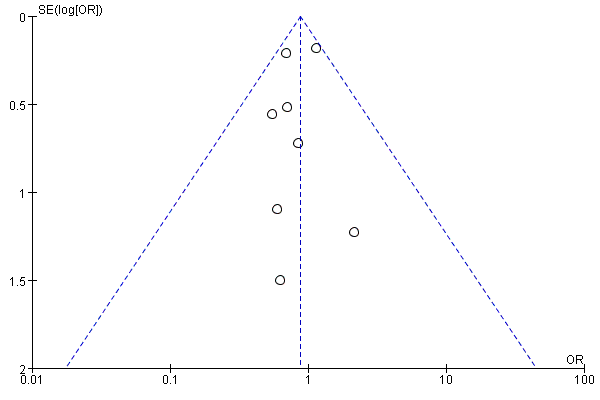
 overall survival and disease-free survival

The funnel plot of pneumonia and lymphedema

The funnel plot of cardiac events and skin toxicities
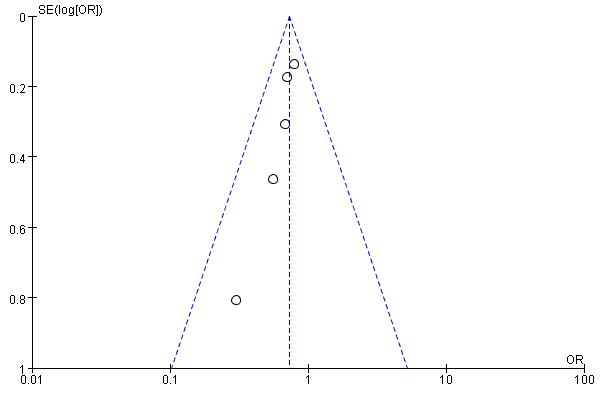

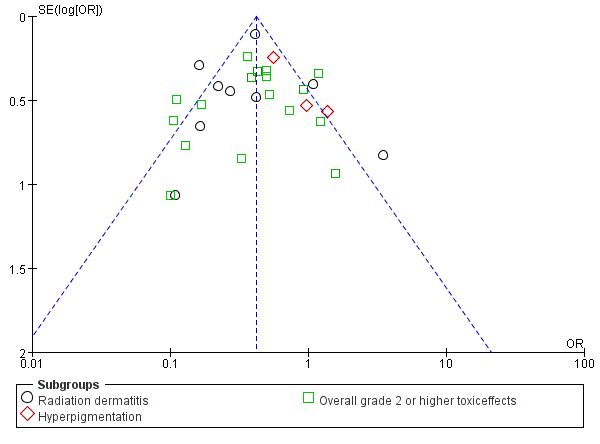

Supplement: Supplementary file 2 — Additional file 2. Funnel plot for other indicators. [file 12885_2024_11918_MOESM2_ESM.docx]
